# Diagnosis of Isocitrate Dehydrogenase-Mutant Astrocytoma in the Subcallosal Gyrus Using T2-Fluid-Attenuated Inversion Recovery Mismatch Sign and Quantitative Magnetic Resonance Relaxometry

**DOI:** 10.7759/cureus.76963

**Published:** 2025-01-05

**Authors:** Yu Tajima, Sayaka Yuzawa, Yonehiro Kanemura, Mishie Tanino, Manabu Kinoshita

**Affiliations:** 1 Department of Neurosurgery, Asahikawa Medical University, Asahikawa, JPN; 2 Department of Diagnostic Pathology, Asahikawa Medical University Hospital, Asahikawa, JPN; 3 Department of Neurosurgery, Biomedical Research, and Innovation, National Hospital Organization (NHO) Osaka National Hospital, Institute for Clinical Research, Osaka, JPN

**Keywords:** brain anatomy, idh mutation, low grade gliomas, mri images, t2-flair mismatch

## Abstract

Astrocytoma, isocitrate dehydrogenase (IDH)-mutant, is one of the intraparenchymal brain tumors, strictly defined by its molecular characteristics. This tumor type is typically found in the frontal, insular, and temporal lobes. Patients harboring this type of tumor benefit the most from aggressive tumor removal compared to other low-grade appearing gliomas. Thus, accurate preoperative diagnosis is crucial in providing these patients with the most efficient and effective treatment strategy. This case study presents a 40-year-old male with an IDH-mutant astrocytoma in the subcallosal gyrus, an unusual location. The diagnosis was aided by the presence of the “T2-FLAIR (fluid-attenuated inversion recovery) mismatch sign,” a key radiological feature of IDH-mutant astrocytomas, accompanied by magnetic resonance (MR) relaxometry that allows quantitative tissue characterization. This case highlights the importance of combining qualitative imaging features, such as the T2-FLAIR mismatch sign, with quantitative data, such as MR relaxometry, for accurate diagnosis, especially in cases with unusual tumor locations.

## Introduction

Astrocytoma, isocitrate dehydrogenase (IDH)-mutant, is one of the intraparenchymal brain tumors, which is strictly defined by its molecular characteristics. The fifth edition of the World Health Organization Classification of Tumors of the Central Nervous System (WHO2021) requires identifying IDH1 or IDH2 mutation without 1p19q co-deletion for diagnosing an astrocytoma [1]. Several retrospective studies suggested that patients harboring an astrocytoma benefit the most from aggressive tumor removal compared to other low-grade appearing tumors, such as radiologically low-grade appearing IDH-wildtype tumors or oligodendrogliomas [2-4]. Thus, accurate preoperative diagnosis is crucial in providing these patients with the most efficient and effective treatment strategy. The T2-fluid-attenuated inversion recovery (FLAIR) mismatch sign is one of the key radiological features, particularly for IDH-mutant astrocytoma [5]. In addition, previous studies have shown that astrocytoma, IDH-mutant, often occurs in frontal, insular, and temporal regions, while oligodendroglioma prefers the medial surface of the frontal lobe [6]. These qualitative imaging features and regional information are complementary to arrive at a correct preoperative diagnosis. Here, the authors report a case of an astrocytoma, IDH-mutant, arising from the subcallosal gyrus. Despite its rare anatomical location [7], the presence of the T2-FLAIR mismatch sign accompanied by a quantitative magnetic resonance (MR) relaxometry map helped to diagnose the pathology before surgery correctly.

## Case presentation

A 40-year-old male was referred to our institution for neuro-oncological treatment as the patient was experiencing a gradual decline in neurocognitive function. A prominent hydrocephalus caused by a space-occupying lesion was observed on computed tomography (CT). Although an intraventricular tumor was initially suspected, careful observation of the image suggested an intraparenchymal tumor arising from the frontal base protruding into the lateral ventricle. Suspecting a glioma​​​​, a comprehensive magnetic resonance image (MRI) study, including MR relaxometry, was scheduled. A 3T MR scanner (Vida; Siemens Healthcare, Erlangen, Germany) was utilized for T1 and T2 mapping.T1-relaxometry was performed by acquiring MP2RAGE images first and converting them into T1-relaxation time maps, while T2-relaxometry was performed by acquiring multi-echo T2-weighted images first and converting them into T2-relaxation time maps. Both relaxometries were performed via Bayesian inference modeling (Olea Nova+; Canon Medical Systems, Tochigi, Japan) [8].

**Figure 1:**
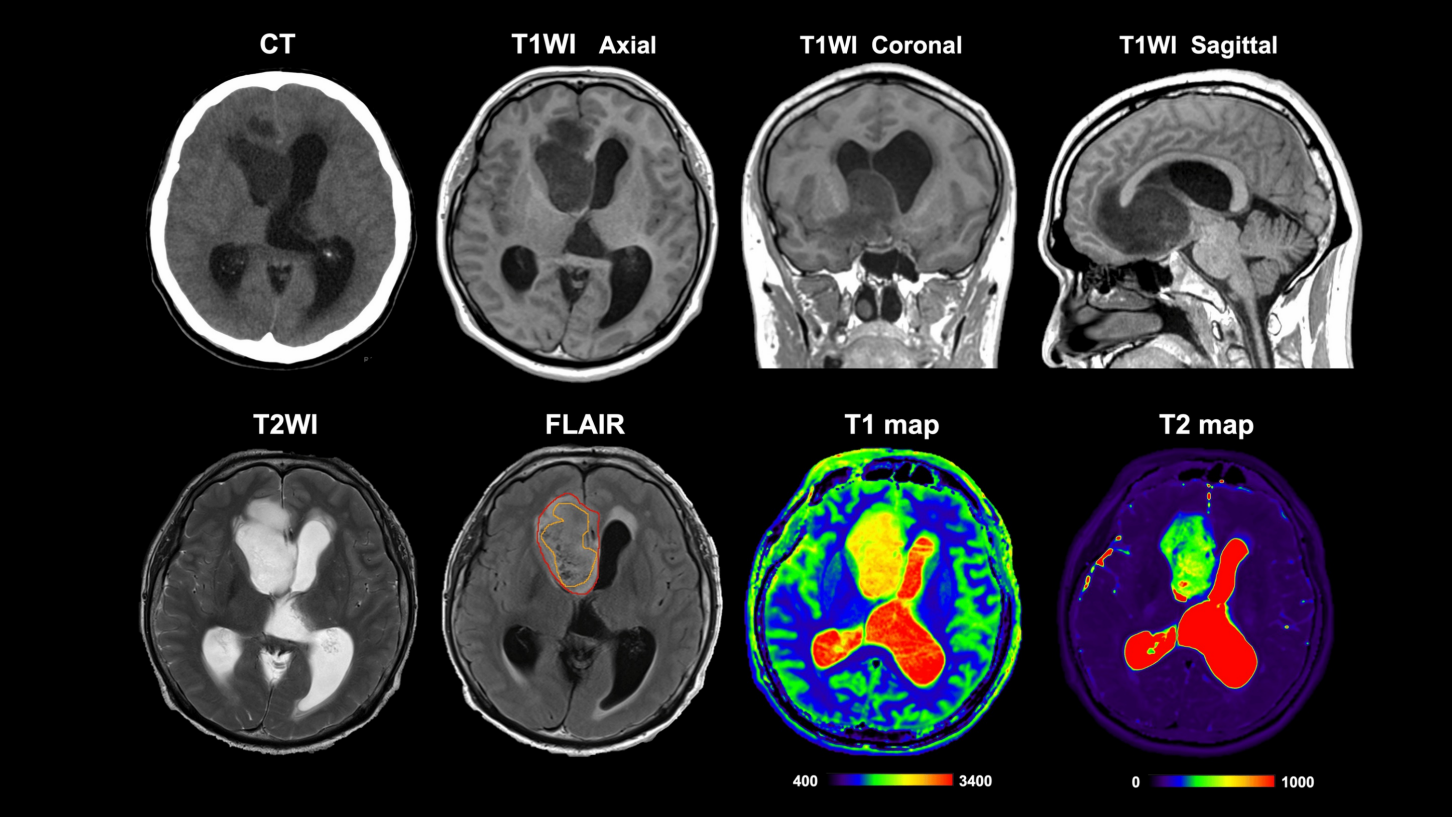
Radiological images before treatment A space-occupying lesion within the right lateral ventricle is observed on computed tomography (CT) and magnetic resonance imaging (MRI) axial images. The sagittal section of the T1-weighted image (T1WI) suggests that the tumor has arisen from the subcallosal gyrus, extending to the anterior cingulate gyrus and putamen. T2-weighted imaging (T2WI) and fluid-attenuated inversion recovery (FLAIR) sequences show a typical "T2-FLAIR mismatch sign." Quantitative magnetic resonance (MR) relaxometry suggests the tumor to be an astrocytoma, isocitrate dehydrogenase (IDH)-mutant. The region of interest (ROI) crafted on FLAIR was placed at the tumor lesion on both T1 and T2 images. The red ROI indicates the entire lesion, while the orange ROI indicates the T2-FLAIR mismatch region.

The lesion seemed to originate from the subcallosal gyrus, infiltrating posteriorly to the caudate and anteriorly to the cingulate gyrus. The lesion appeared hyperintense on T2-weighted images (T2WI) and hypointense with a hyperintense rim on FLAIR, which fulfills the T2-FLAIR mismatch sign criteria. The entire lesion's mean T1- and T2 relaxation times were 2257 and 372 msec, respectively, while those of the T2-FLAIR mismatch were 2453 and 443 msec, respectively.Astrocytoma’s T1- and T2- relaxation times are reported to range from 1000 to 3000 msec with a mean of 2047 msec [9] and 200 to 750 msec, with a median of 400 msec, respectively [8]. On the other hand, T1- and T2- relaxation times ​​​​​of IDH-wildtype tumors are from 1000 to 2500 msec and from 100 to 200 msec, and those of oligodendrogliomas range from 1000 to 3500 msec and from 100 to 400 msec [8]. Despite its unusual anatomical location, qualitative and quantitative imaging features strongly suggested an IDH-mutant astrocytoma arising from the subcallosal gyrus (Figure 1).

**Figure 2:**
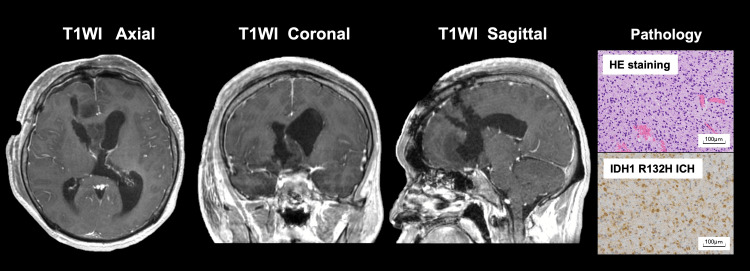
Radiological images after surgery accompanied by pathological images Seventy percent of the tumor volume was resected via the trans-superior frontal gyrus route with improved hydrocephalus. Hematoxylin and eosin (HE) staining showed diffuse proliferation of atypical astrocytic cells, and IDH1 R132H immunohistochemistry (ICH) confirmed IDH1 mutation. Pathological photographs are taken at 20x magnification. The final diagnosis was astrocytoma, IDH-mutant, CNS WHO grade 3.

Surgical removal of the tumor was performed via the trans-superior frontal gyrus route. Post-surgical MRI confirmed a 70% tumor removal with a radiological improvement of the hydrocephalus. IDH1 R132H mutation, but not the telomerase reverse transcriptase (TERT)​​​​​​​ promotor mutation, was identified by Sanger sequencing. The O(6)-methylguanine-DNA methyltransferase (MGMT)​​​​​​​ promoter was identified to be methylated by quantitative methylation-specific PCR after bisulfite modification of genomic DNA with a threshold of ≥1%. Homozygous deletion of CDKN2A/B was not observed by Multiplex Ligation-dependent Probe Amplification (MLPA) using the SALSA MLPA kit probemix P088-C2 for 1p/19q analysis (MRC Holland, Amsterdam, Netherlands). The third author performed all genetic analyses at the National Hospital Organization (NHO)​​​​​​​ Osaka National Hospital. The final pathological diagnosis was astrocytoma, IDH-mutant, CNS WHO grade 3. The patient is scheduled to undergo a second surgery aiming for an extent of resection greater than 90%, as it has been discussed that the extent of resection could have a significant prognostic impact on astrocytoma, IDH-mutant, CNS WHO grade 3 (Figure 2) [10].

## Discussion

Accurate pre-surgical diagnosis is key to achieving the most effective treatment for any disease. Many attempts have been made to fulfill this goal in neuro-oncology. As neuroimaging plays an essential role in characterizing neurological disorders in the current era, various radiological signatures have been proposed to assist non-invasive diagnosis of brain tumors. The authors and others have already reported spatial preferences of each glioma subtype, showing that astrocytoma, IDH-mutant, most often arises at the frontal to temporal regions [6]. This information can be helpful in the process of diagnosing glioma subtypes. However, a small fraction of tumors can also occur outside the preferred zones, such as the occipital lobe [11], complicating the diagnostic process.

The T2-FLAIR mismatch sign is a qualitative radiological feature reported to be highly specific for astrocytoma, IDH-mutant. This imaging feature was first discovered by reviewing The Cancer Imaging Archive (TCIA) low-grade glioma dataset, followed by a New York University cohort validation [5]. While the specificity was first reported to be 100%, some investigators started to adopt more “relaxed” criteria, which lowered its specificity to some extent later on [12,13]. Its relatively low sensitivity has always been an issue since its proposal. In fact, TCIA’s image acquisition protocol for FLAIR differed substantially from one patient to another [14,15]. To address these issues, a quantitative approach has also been attempted. Considering that FLAIR is designed to attenuate MR signals originating from tissues within a specific range of T1-relaxation time, a direct relaxometry of the tumor tissue could circumvent issues derived from qualitative radiological assessment. Several studies, including this study, have shown that astrocytoma, IDH-mutant, falls within the specific range of T1- and T2-relaxation time [8,9]. The presented case harbored an intraparenchymal tumor at a location somewhat unfamiliar as an astrocytoma. However, both qualitative and quantitative assessments strongly suggested otherwise, leading to a treatment strategy focused on radical tumor removal.

While MR relaxometry is a promising quantitative MRI technique, its relatively long scan time is a drawback. However, rapid advancements in MRI technology are anticipated to incorporate more quantitative assessments into glioma patient care. For instance, MR fingerprinting (MRF) can acquire MR relaxometry data with a scan time of approximately 5 minutes, while the MR relaxometry of the presented case requires 20 minutes. This kind of technological advancement could significantly impact pre-surgical glioma patient care.

## Conclusions

We reported an IDH-mutant astrocytoma arising from the subcallosal gyrus, an uncommon location. The correct pre-surgical diagnosis was achieved by utilizing the T2-FLAIR mismatch sign and quantitative MR relaxometry.
